# Ritonavir-Boosted Darunavir Plus Two Nucleoside Reverse Transcriptase Inhibitors versus Other Regimens for Initial Antiretroviral Therapy for People with HIV Infection: A Systematic Review

**DOI:** 10.1155/2017/2345617

**Published:** 2017-09-26

**Authors:** Tatevik Balayan, Hacsi Horvath, George W. Rutherford

**Affiliations:** ^1^Global Health Sciences, University of California, San Francisco, San Francisco, CA, USA; ^2^School of Public Health, American University of Armenia, Yerevan, Armenia

## Abstract

**Background:**

Darunavir is a second-generation protease-inhibitor used with ritonavir (DRV/r) and two nucleoside reverse-transcriptase inhibitors as an option in first-line antiretroviral treatment (ART).

**Methods:**

We systematically reviewed randomized controlled trials (RCTs) of DRV/r versus other regimens in patients initiating ART. We searched five bibliographic databases and other key resources. We had no language limitations. We assessed bias risk with the Cochrane tool and used GRADE to assess evidence quality. We report findings in terms of risk ratio (RR) with 95% confidence intervals (CI).

**Findings:**

Three RCTs met inclusion criteria. In plasma viral load suppression, DRV/r outperformed ritonavir-boosted lopinavir at 48 weeks (RR 1.13, 95% CI 1.03–1.25), 96 weeks (RR 1.11, 95% CI 1.02–1.21), and 192 weeks (RR 1.20, 95% CI 1.07–1.35). DRV/r was similar to dolutegravir at 48 weeks (RR 0.96, 95% CI 0.87–1.06) but less effective at 96 weeks (RR 0.84, 95% CI 0.75–0.93). At 96 weeks, DRV/r underperformed raltegravir (RR 0.94, 95% CI 0.88–0.99) but was similar to ritonavir-boosted atazanavir (RR 1.02, 95% CI 0.96–1.09). Overall bias risk was moderate. Evidence quality was also moderate.

**Interpretation:**

Initial ART regimens using DRV/r should be considered in future World Health Organization guidelines.

## 1. Introduction

Darunavir (DRV) is a once-daily second-generation protease-inhibitor [1, 2] that is administered with low-dose ritonavir (DRV/r) and two nucleoside reverse transcriptase inhibitors (NRTI) for treatment of HIV infection. In vitro studies have shown that resistance to DRV develops much more slowly and that it has a higher genetic barrier for the development of resistance relative to current protease inhibitors [3]. DRV has a very low resistance profile [3], requires boosting with ritonavir, and is used in combinations with two NRTIs, such as abacavir (ABC) + lamivudine (3TC) or tenofovir (TDF) + emtricitabine (FTC).

DRV/r + two NRTIs is the third option in the United States (US) Department of Health and Human Services' and the European AIDS Clinical Society's six recommended initial regimens for antiretroviral-naïve HIV-infected patients [4, 5]. The British HIV Medical Association has also recommended it as one of six third-line agents to be used with a two-drug NRTI backbone [6]. In contrast World Health Organization (WHO) guidelines only recommend DRV/r with two NRTIs as second- and third-line regimens for adults and adolescents who have failed initial therapy [7]. Different studies have shown that DRV/r combination therapy is less expensive than other combination therapies such as ritonavir-boosted lopinavir (LPV/r) [8] and ritonavir-boosted atazanavir (ATV/r) [8] but less cost effective compared to dolutegravir (DTG) [9] and raltegravir (RAL) [10].

In this paper, we systematically review the efficacy and safety of DRV/r in combination with two NRTIs compared to the current WHO standard regimens of efavirenz (EFV), DTG, LPV/r, ATV/r, and RAL with two NRTIs.

## 2. Methods

We used Cochrane Collaboration methods throughout the review process [11]. We followed the Preferred Reporting Items for Systematic Reviews and Meta-Analyses (PRISMA) guidance in reporting our results [12]. Before beginning our review, we registered its protocol in the PROSPERO online registry (registration number CRD42016040058).

### 2.1. Search Methods

We used a comprehensive search strategy to identify all relevant studies. We searched the Cochrane Central Register of Controlled Trials, Embase, Literatura Latino Americana em Ciências da Saúde (LILACS), PubMed, and Web of Science. In our search strategy, we included Medical Subject Heading (MeSH) or other database-specific indexing terms, as well as a range of relevant keywords. Searches captured all records up to the search date (June 9, 2016). We modified our core PubMed search strategy as needed for each database. See Supplement 1 for our PubMed search strategy, modified and adapted as needed for use in the other databases (https://doi.org/10.1155/2017/2345617).

We searched available conference abstracts from three major HIV/AIDS conferences (Conference on Retroviruses and Opportunistic Infections, the International AIDS Conference, and the International AIDS Society Conference on HIV Pathogenesis, Treatment and Prevention). We searched the clinical trials registry (clinicaltrials.gov) of the US National Institutes of Health to identify ongoing trials, and any others we might have missed in searches of the peer-reviewed literature. We also examined the reference lists of our included studies and other highly relevant studies. We had no restrictions by language or publication status.

### 2.2. Inclusion and Exclusion Criteria

We included RCTs that compared clinical and laboratory outcomes in HIV-1-infected, ART-naïve adults and adolescents starting regimens of DRV/r plus two NRTIs with those starting regimens of EFV, ATV/r, LPV/r, DTG, and RAL plus two NRTIs. We excluded nonrandomized studies and studies in which participants were ART-experienced. All studies found were written in English.

### 2.3. Data Extraction

We imported search results into bibliographic citation management software (EndNote X7, Thomson Reuters, New York, New York, USA). One author (Hacsi Horvath) removed duplicate records and, by reviewing article titles, excluded all clearly irrelevant records. Following this, two authors (George W. Rutherford and Tatevik Balayan) each independently reviewed the titles, abstracts, and descriptor terms of all remaining citations. For all records deemed potentially eligible for inclusion, we obtained full-text articles. Applying the inclusion criteria, George W. Rutherford and Tatevik Balayan reviewed these articles and determined those that were indeed eligible for inclusion. In the event of disagreements during the screening process, we planned to resolve them through discussion. If necessary, a neutral third person would have contributed to the decision.

Two authors (Tatevik Balayan and Hacsi Horvath) each independently extracted data from all trials meeting inclusion criteria and separately entered these data into standardized data extraction forms. Extracted data in each author's form were then compared and were found to match.

### 2.4. Risk of Bias Assessment

We assessed risk of bias in the included RCTs by using the Cochrane Collaboration's tool [11]. This instrument assesses six domains of bias risk: sequence generation, allocation concealment, blinding, incomplete outcome data, selective outcome reporting, and other potential biases.

### 2.5. Data Synthesis and Analysis

We calculated the relative risk (RR) for dichotomous outcomes and mean difference (MD) for continuous outcomes, each with 95% confidence intervals (CI). Where appropriate and possible, we had planned to pool data across studies and estimate summary effect sizes, using a Mantel-Haenszel fixed-effects meta-analytic model. Had we been able to conduct meta-analyses, we would have used Review Manager 5.3 (Nordic Cochrane Centre, Copenhagen, Denmark).

We planned to present estimates of heterogeneity in pooled data, as determined by the *I*^2^ statistic. *I*^2^ statistic estimates attempt to show the percentage of variability in effect estimates that arise from statistical heterogeneity and not from chance. If we had pooled data and found high statistical heterogeneity, we would have investigated it through sensitivity analyses.

We assessed the quality of evidence for each outcome across all studies by using the GRADE methodology [13]. “Quality of evidence” in GRADE is “the extent of our confidence that the estimates of effect are correct" [13]. GRADE rates evidence quality at four levels: high, moderate, low, or very low. RCT data for a given outcome are considered at the outset to provide high quality evidence. Observational study data are deemed at the outset to provide low quality evidence. Depending on specific circumstances, evidence quality can be downgraded for high risk of bias, indirectness, inconsistency of effect estimates, statistical imprecision, or high risk of publication bias. In RCT data that have been downgraded, it can also potentially be graded up if there is a large magnitude of effect, if plausible confounding would increase confidence in an estimated effect, or if a dose-response gradient is observed. If there has been no downgrading, observational study data can potentially be graded up for the same reasons.

## 3. Results

**Figure 1:**
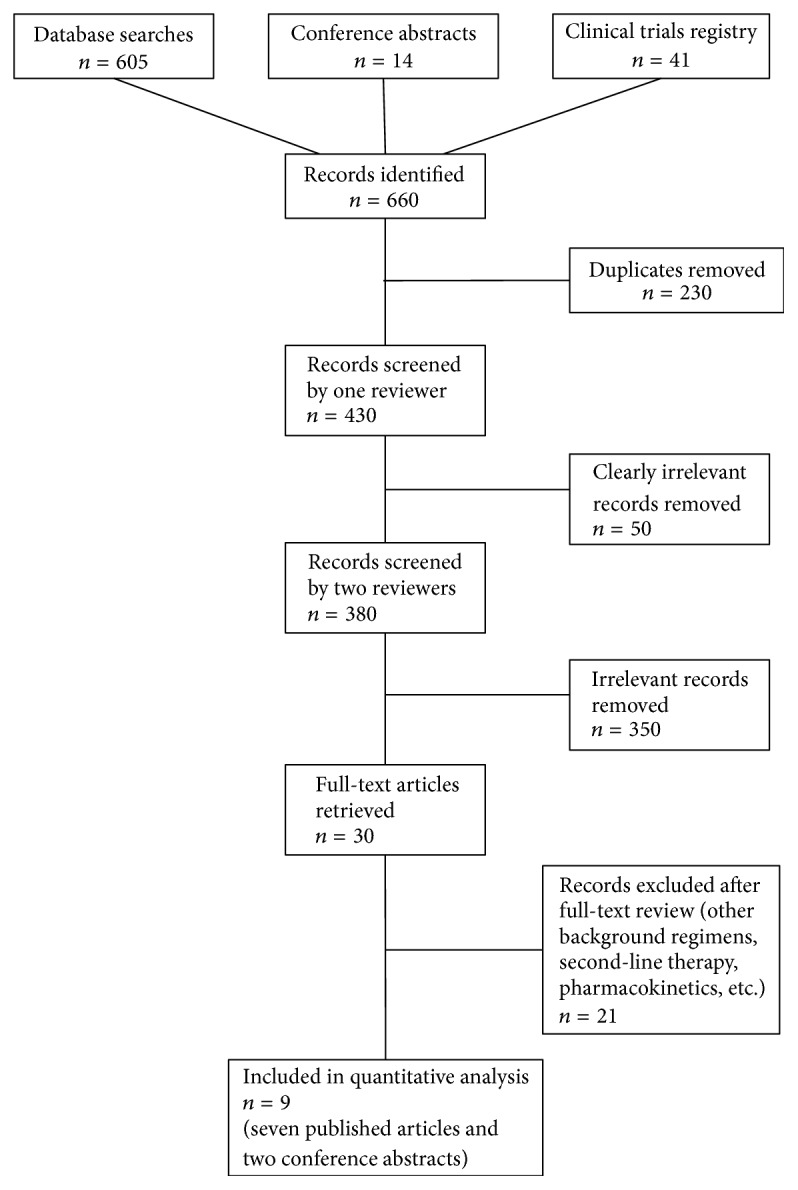
PRISMA flowchart.

We initially identified 660 articles (605 from bibliographic databases, 14 from conference abstracts, and 41 from registered trials). After removing 230 duplicate records and 50 clearly irrelevant records, we independently reviewed 380 titles and abstracts and excluded 350 clearly irrelevant records. We selected 30 records for full-text review. We then excluded 21 studies reporting results of other background regimens, second-line therapy, pharmacokinetics, and other topics (Figure 1).

Three trials reported in eight published articles and one conference abstract met our inclusion criteria. All studies were RCTs (Table 1). The trials were conducted in Argentina, Australia, Austria, Belgium, Canada, Chile, Costa Rica, Denmark, France, Germany, Greece, Guatemala, Malaysia, Mexico, Panama, Puerto Rico, Russia, Singapore, South Africa, Spain, Switzerland, Taiwan, Thailand, the United Kingdom, and the United States of America. Overall, there were 2,986 participants who were randomized. The first trial (ARTEMIS) was a two-arm Phase III trial that compared DRV/r + TDF/FTC (*N* = 343) with LPV/RTV + TDF/FTC (*N* = 346) [14–17]. The second trial (FLAMINGO) was a Phase IIIb two-arm trial that compared DRV/r + ABC/3TC or TDF/FTC with DTG + ABC/3TC or TDF/FTC in 488 ART-naïve patients [18, 19]. The third trial (ACTG 5257) was Phase III three-arm trial that compared DRV/RTV + FTC/TDF with ATV/RTV + FTC/TDF and RAL + FTC/TDF [20–22]. We identified no trials that compared DRV/r to EFV. ARTEMIS contributed 343 patients to the DRV/r arm and 346 to the LPV/r arm [14–17]; FLAMINGO contributed 245 patients to the DRV/r arm and 243 to the DTG arm [18, 19]; and ACTG 5272 contributed 601 patients to the DRV/r arm, 603 to the RAL arm, and 605 to the ATV arm [20–22].

Given the important differences among intervention and comparator regimens in the included trials we did not pool data in meta-analysis for any outcome.

### 3.1. Viral Suppression

The primary endpoint of all three trials was viral suppression to <50 copies/mL at 48, 96, and 192 weeks (Table 2). In the ARTEMIS trial, the DRV/r-containing regimen was superior to the LPV/r-containing regimen at 48 weeks, 96 weeks, and 192 weeks (RR 1.13, 95% CI 1.03–1.25; RR = 1.11, 95% CI 1.02–1.21; and RR 1.20, 95% CI 1.07–1.35, resp.) [14, 15]. However, among participants with baseline HIV RNA levels of >100,000 copies/mL, virologic response rates were lower in the LPV/r arm than in the DRV/r arm (ARTEMIS). In the FLAMINGO trial, the rate of virologic suppression at 48 weeks was no different in the DRV/r arm and the DTG arm (RR 0.96, 95% CI 0.87–1.06); however by 96 weeks viral suppression was significantly higher in the DTG arm compared to the DRV/r arm (RR 0.84, 95% CI 0.75–0.93). The excess failure observed in the DRV/r group was primarily related to a higher rate of virologic failure among those with a viral load > 100,000 copies/mL and secondarily due to more drug discontinuations in the DRV/r group [19]. In ACTG 5257, which was a three-way comparison of DRV/r, ATV/r, and RAL, 96-week virologic suppression in the DRV/r arm was similar to that in the ATV/r arm (RR 1.02, 95% CI 0.96–1.09) but inferior to RAL (RR 0.94, 95% CI 0.88–0.99). However, more participants in the ATV/r group discontinued treatment because of adverse events [21].

**Figure 2:**
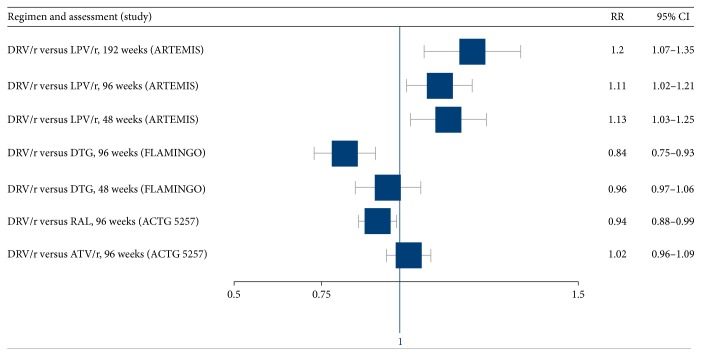
DRV/r-based regimens: efficacy in viral suppression, sorted by regimen.

**Figure 3:**
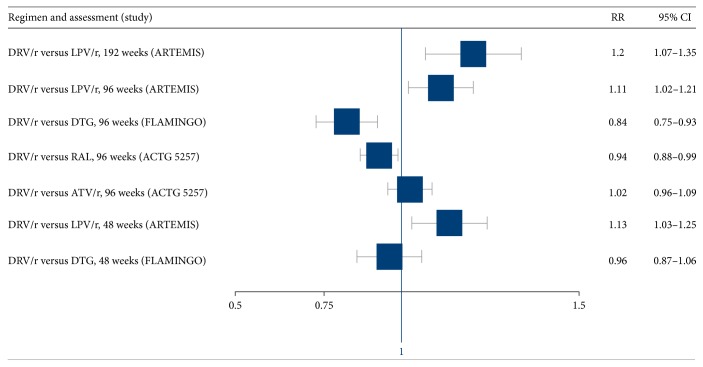
DRV/r-based regimens: efficacy in viral suppression, sorted when outcome was assessed.

Figure 2 depicts viral suppression outcomes, sorted by regimen.  Figure 3 also depicts these outcomes, sorted by length of follow-up.

### 3.2. Mortality

There were no significant differences in mortality in any trial between patient arms randomized to DRV/r and those randomized to other regimens.

DRV/r versus LPV/r: at 48 weeks, one patient receiving DRV/r had died, compared to three deaths in patients receiving LPV/r (RR 0.34, 95% CI 0.04–3.22). No additional patients in either arm had died at the 96-week assessment. At 192 weeks, a total of four patients receiving DRV/r and seven receiving LPV/r had died (RR 0.58, 95% CI 0.17–1.95).

DRV/r versus RAL or ATV/r: at 96 weeks, 13 patients receiving DRV/r had died, compared to six deaths in patients receiving RAL (RR 2.17, 95% CI 0.83–5.68) and 10 deaths in patients receiving ATV/r (RR 1.31, 95% CI 0.58–2.96).

DRV/r versus DTG: at 96 weeks, no patients had died in either arm of the trial comparing DRV/r with DTG.

### 3.3. Severe Adverse Events

In the ARTEMIS trial risk of serious adverse events was lower in the DRV/r arm than in the LPV/r arm at 48 and 96 weeks (RR 0.62, 95% CI 0.38–0.99; RR 0.62, 95% CI 0.42–0.93, resp.) but similar in both arms at 192 weeks (RR 0.77, 95% CI 0.56–1.06). In the FLAMINGO trial risk of serious adverse events was lower in the DRV/r arm than in the DTG arm at 48 weeks (RR 0.50, 95% CI 0.26–0.94) but higher at 96 weeks (RR 1.70, 95% CI 1.02–2.83). In the ACTG 5257 trial the risk of serious adverse events at 96 weeks was higher in DRV/r arm compared to ATV/r (hyperbilirubinaemia) (RR 1.64, 95% CI 1.49–1.80) but similar in the DRV/r and RAL arms (RR 1.05, 95% CI 0.99–1.12).

For the viral suppression, mortality, and SAE outcomes, see Table 2.

### 3.4. Immunologic Recovery

The principal secondary outcome was CD4 recovery (reaching and maintaining a higher CD4 cell count compared to baseline data). ARTEMIS contributed data to 48-, 96-, and 192-week outcomes; FLAMINGO contributed to 48- and 96-week outcomes; ACTG 5257 contributed to 96-week outcomes (Table 3). No trial reported confidence intervals for CD4 count change so it was not possible to judge the significance of the difference. Considering only reported point estimates, immune recovery was less robust among patients taking the DRV/r-based regimen in ARTEMIS study at 48, 96, and 192 weeks. The same is true for FLAMINGO at 48 and 96 weeks and ACTG 5257 at 96 weeks; see Table 3.

### 3.5. Risk of Bias in the Included Studies

**Figure 4:**
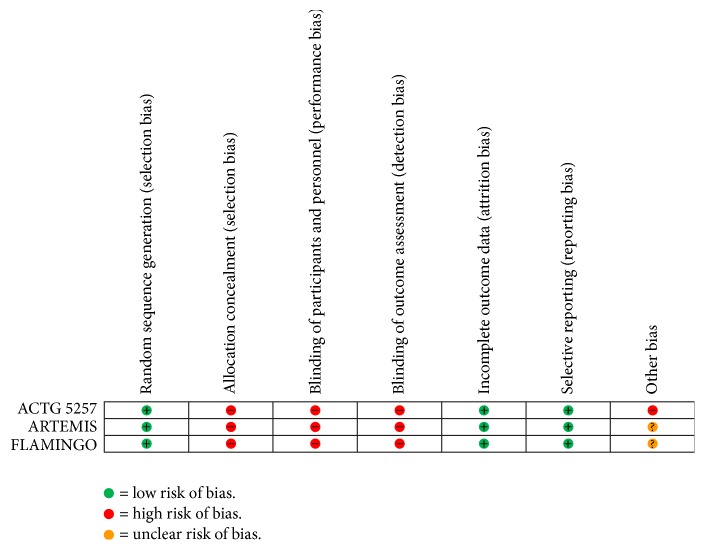
Risk of bias. Review authors' judgments about each risk of bias item for included studies.

In the ARTEMIS and FLAMINGO trials, methods for sequence generation were adequate, with centralized, computer-based procedures used to randomize patients within baseline CD4 and viral load strata in ARTEMIS and within viral load strata in FLAMINGO (Figure 4). In the ACTG 5257 trial, randomization used permuted blocks stratified according to the viral load strata with balancing by institution. All trials were open label. Allocation was not concealed, and none of the studies was blinded. However, in all three trials the outcomes were biomedical and not susceptible to detection bias. There was evidence of incomplete outcome ascertainment due to differential loss to follow-up in the longer series, for instance, 24.8% of DRV/r arm participants and 32.9% of LPV/r arm participants left the trial by week 192. There was no evidence of selective reporting. All three trials were sponsored by pharmaceutical companies, although we detected no obvious problems attributable to industry involvement. Supplement 2 provides a detailed assessment of bias risk in each trial.

### 3.6. Quality of the Evidence

For the key virologic suppression outcomes, evidence quality was high for the DRV/r versus LPV/r comparison at 48 weeks but moderate at 96 and 192 weeks, graded down for risk of bias (high attrition). For the DRV/r versus DTG comparison the evidence quality for virologic suppression was high at both 48 and 96 weeks, as it was for the DRV/r versus ATV/r comparison and the DRV/r versus RAL at 96 weeks. The evidence quality was much less robust for the mortality outcomes and was rated as low or very low across all four comparisons owing to serious imprecision due to few events and in the DRV/r versus LPV/r comparison owing to risk of bias (high attrition). The evidence quality for severe adverse events was low or very low owing to few events and, again, high attrition in the trial of DRV/r versus LPV/r. See Supplement 3 for our complete GRADE evidence profile analysis of evidence quality.

## 4. Discussion

We found that DRV/r-containing regimens were associated with a greater proportion of patients being virologically suppressed up to 192 weeks after initiation of therapy compared to LPV/r-based regimens. However, we also found out that DRV/r-based regimens were inferior to DTG- and RAL-based regimens at 96 weeks. There was no difference between DRV/r and ATV/r regimens in terms of virologic suppression.

Participants in the DRV/r-containing regimens were almost three times less likely to discontinue their original regimen because of adverse events or to die than those in the LPV/r arms by 192 weeks. However, discontinuation because of adverse events or death was about 2 times higher among those in DRV/r regimen than DTG regimen, about three times higher compared with the ATV/r arm, and five times higher compared with the RAL regimen.

As with any systematic review our study is limited by the sensitivity of our search and our ability to identify studies that meet our inclusion criteria. We attempted to minimize this risk by comprehensively searching four key databases and hand searching abstracts from three major conferences as well as the bibliographies not only of included articles but also of review articles. Secondly, the three trials on which our conclusions are based were conducted across all over the world, but only South Africa was included from Africa; given that the large majority of HIV-infected patients are in Africa, this may somewhat limit the generalizability of our findings. Finally, we used the GRADE system to rate the quality of this literature. A recent evaluation of how GRADE is being used at WHO found some remaining challenges [23], but it has emerged as the gold standard for guideline development at WHO [24] and is required by the Guideline Review Committee, which approves all new guidelines.

## 5. Conclusions

We found three RCTs that directly compared DRV/r with other regimens for initial treatment of HIV infection in adults and adolescents. DRV/r appears to be superior to LPV/r in terms of durable viral suppression and immunologic recovery, inferior to DTG and RAL and similar to ATV/r. DRV/r-containing regimens should be considered in future international guidelines for initial therapy of HIV infections, but its utility has likely been eclipsed by better performing integrase inhibitor regimens.

## Supplementary Material

Supplement 1: PubMed search strategy, modified and adapted as needed for use in the other databases.
Supplement 2: Detailed risk of bias assessment.
Supplement 3: GRADE evidence profile.

## Figures and Tables

**Table 1:** Characteristics of included studies.

Study	Clinicaltrials.gov identifier	Setting	Participants	Intervention	Comparator
ARTEMIS	NCT00258557	Argentina, Australia, Austria, Belgium, Canada, Chile, Costa Rica, Denmark, France, Germany, Greece, Guatemala, Malaysia, Mexico, Panama, Puerto Rico, Russia, Singapore, South Africa, Spain, Switzerland, Taiwan, Thailand, United Kingdom, United States	689	DRV/r + TDF/FTC	LPV/r + TDF/FTC

FLAMINGO	NCT00951015	France, Germany, Italy, Romania, Russia, Spain, Switzerland, United States	286	DRV/r + ABC/3TC or TDF/FTC	DTG + ABC/3TC or TDF/FTC

ACTG 5272	NCT00811954	Puerto Rico, United States	1809	DRV/RTV + FTC/TDF	ATV/r + FTC/TDF RAL + FTC/TDF

DRV/r, ritonavir-boosted darunavir; DTG, dolutegravir; ABC, abacavir; 3TC, lamivudine; FTC, emtricitabine; TDF, tenofovir; ATV/r, ritonavir-boosted atazanavir; RAL, raltegravir.

**Table 2:** Quantitative data for trial outcomes (dichotomous).

DRV/r versus LPV/r (ARTEMIS)	Events, DRV/r	Total, DRV/r	Events, LPV/r	Total, LPV/r	RR (95% CI)
Mortality (192 weeks)	4	343	7	346	0.58 (0.17–1.95)
Mortality (96 weeks)	1	343	3	346	0.34 (0.04–3.22)
Mortality (48 weeks)	1	343	3	346	0.34 (0.04–3.22)
≥1 SAE (192 weeks)	55	343	72	346	0.77 (0.56–1.06)
≥1 SAE (96 weeks)	34	343	55	346	0.62 (0.42–0.93)
≥1 SAE (48 weeks)	25	343	41	346	0.62 (0.38–0.99)
PVL < 50 copies/mL (192 weeks)	236	343	198	346	1.20 (1.07–1.35)
PVL < 50 copies/mL (96 weeks)	271	343	246	346	1.11 (1.02–1.21)
PVL < 50 copies/mL (48 weeks)	254	343	226	346	1.13 (1.03–1.25)

DRV/r versus DTG (FLAMINGO)	Events, DRV/r	Total, DRV/r	Events, DTG	Total, DTG	RR (95% CI)

≥1 SAE (96 weeks)	36	245	21	243	1.70 (1.02–2.83)
≥1 SAE (48 weeks)	13	245	26	243	0.50 (0.26–0.94)
PVL < 50 copies/mL (96 weeks)	164	245	194	243	0.84 (0.75–0.93)
PVL < 50 copies/mL (48 weeks)	186	245	192	243	0.96 (0.87–1.06)

DRV/r versus RAL (ACTG 5257)	Events, DRV/r	Total, DRV/r	Events, RAL	Total, RAL	RR (95% CI)

Mortality (96 weeks)	13	601	6	603	2.17 (0.83–5.68)
Elevated blood bilirubin (96 weeks)	466	601	444	603	1.05 (0.99–1.12)
PVL < 50 copies/mL (96 weeks)	461	601	494	603	0.94 (0.88–0.99)

DRV/r versus ATV/r (ACTG 5257)	Events, DRV/r	Total, DRV/r	Events, ATV/r	Total, ATV/r	RR (95% CI)

Mortality (96 weeks)	13	601	10	605	1.31 (0.58–2.96)
Elevated blood bilirubin (96 weeks)	466	601	286	605	1.64 (1.49–1.80)
PVL < 50 copies/mL (96 weeks)	461	601	455	605	1.02 (0.96–1.09)

RR, risk ratio; PVL, plasma viral load; SAE, severe adverse event; CI, confidence interval; DRV/r, ritonavir boosted darunavir-based regimen; DTG, dolutegravir-based regimen; LPV/r, ritonavir boosted lopinavir-based regimen; ATV/r, ritonavir boosted atazanavir-based regimen; RAL, raltegravir-based regimen.

**Table 3:** Quantitative data for trial outcomes (continuous).

DRV/r versus LPV/r (ARTEMIS)	Mean change, DRV/r	Total, DRV/r	Mean change, LPV/r	Total, LPV/r
Immunologic recovery (192 weeks)	+258 cells/*µ*L	343	+263 cells/*µ*L	346
Immunologic recovery (96 weeks)	+171 cells/*µ*L	343	+188 cells/*µ*L	346
Immunologic recovery (48 weeks)	+137 cells/*µ*L	343	+141 cells/*µ*L	346

DRV/r versus DTG (FLAMINGO)	Mean change, DRV/r	Total, DRV/r	Mean change, DTG	Total, DTG

Immunologic recovery (96 weeks)	+250 cells/*µ*L	245	+260 cells/*µ*L	243
Immunologic recovery (48 weeks)	+210 cells/*µ*L IQR 110–290	245	+210 cells/*µ*L IQR 120–350	243

DRV/r versus RAL (ACTG 5257)	Mean change, DRV/r	Total, DRV/r	Mean change, RAL	Total, RAL

Immunologic recovery (96 weeks)	+256 cells/*µ*L	601	+288 cells/*µ*L	603

DRV/r versus ATV/r (ACTG 5257)	Mean change, DRV/r	Total, DRV/r	Mean change, ATV/r	Total, ATV/r

Immunologic recovery (96 weeks)	+256 cells/*µ*L	601	+280 cells/*µ*L	605

IQR, interquartile range; DRV/r, ritonavir-boosted darunavir-based regimen; DTG, dolutegravir-based regimen; LPV/r, ritonavir-boosted lopinavir-based regimen; ATV/r, ritonavir-boosted atazanavir-based regimen; RAL, raltegravir-based regimen.
