# Supplementary material for: Ritonavir-Boosted Darunavir Plus Two Nucleoside Reverse Transcriptase Inhibitors versus Other Regimens for Initial Antiretroviral Therapy for People with HIV Infection: A Systematic Review
Source: AIDS Res Treat. 2017 Sep 26;2017:2345617. doi: 10.1155/2017/2345617 (PMC5634582; doi:10.1155/2017/2345617)
Supplement: Supplementary file 1 — Supplement 1: PubMed search strategy, modified and adapted as needed for use in the other databases. Supplement 2: Detailed risk of bias assessment. Supplement 3: GRADE evidence profile. [file 2345617.f1.zip › mat.2345617.v2/S1_PRISMA.docx]

**Supplement 1.** PubMed search strategy, modified and adapted as needed for use in the other databases

| **Search** | **PubMed query** |
| --- | --- |
| **#5** | **Search #1 AND #2 AND #3 AND #4** |
| #4 | Search randomized controlled trial[pt] OR randomized controlled trials[mh] OR random allocation[mh] OR controlled clinical trial[pt] OR randomized[tw] OR randomised[tw] OR randomly[tw] OR random*[tw] OR trial[tiab] OR groups[tiab] OR Cohort Studies[mh] OR cohort[tw] OR cohorts[tw] |
| #3 | Search Treatment-naïve[tw] OR ART-naïve[tw] OR ARV-naïve[tw] OR naïve[tw] OR naïfs[tw] OR first-line[tw] OR firstline[tw] OR initial[tw] OR initiating[tw] OR initiated[tw] |
| #2 | Search HIV Protease Inhibitors[mh] OR HIV Infections/drug therapy[mh] OR Antiretroviral Therapy, Highly Active[mh] OR Anti-Retroviral Agents[mh] OR protease inhibitor*[tw] OR “second generation”[tw] OR “2^nd^ generation”[tw] OR antiretroviral[tw] OR anti-retroviral[tw] OR anti-HIV*[tw] OR anti-AIDS[tw] |
| #1 | Search Darunavir[tw] OR DRV[tw] DRV/r OR TMC114[tw] OR TMC-114[tw] OR Prezista[tw] OR Darunavir[mh] |

Legend: [mh] = Medical Subject Heading (MeSH) term. [pt] = publication type. [tw] = text word. [tiab] = title or abstract. *= “wildcard”; any character or sequence of characters may follow.
