# Supplementary material for: Ritonavir-Boosted Darunavir Plus Two Nucleoside Reverse Transcriptase Inhibitors versus Other Regimens for Initial Antiretroviral Therapy for People with HIV Infection: A Systematic Review
Source: AIDS Res Treat. 2017 Sep 26;2017:2345617. doi: 10.1155/2017/2345617 (PMC5634582; doi:10.1155/2017/2345617)
Supplement: Supplementary file 1 — Supplement 1: PubMed search strategy, modified and adapted as needed for use in the other databases. Supplement 2: Detailed risk of bias assessment. Supplement 3: GRADE evidence profile. [file 2345617.f1.zip › mat.2345617.v2/S2_Risk of bias.docx]

**Supplement 2.** Detailed risk of bias assessment

| **Risk of bias: ARTEMIS** | **Judgment** | **Rationale** |
| --- | --- | --- |
| Random sequence generation (selection bias) | Low | Randomization (1:1) was done by predefined randomization list and central randomization system was used. Patients were stratified at screening by plasma RNA (<100,000 copies/mL, ≥100,000 copies/mL) and NRTI selection. |
| Allocation concealment (selection bias) | Low | Allocation was not concealed, but risk of bias was low as all outcomes were biomedical. |
| Blinding of participants and personnel (performance bias) | High | The trial was open-label; patients and physicians were aware of group allocations. Risk of bias was low as all outcomes were biomedical. |
| Blinding of outcome assessment (detection bias) | Unclear | Study was open label. There is no information about the blinding of outcome assessors. |
| Incomplete outcome data (attrition bias) | Low | Loss to follow-up was light (5.2% -9.2%) at 96 weeks of the study and investigators described it adequately. |
| Selective reporting (reporting bias) | Low | Reported study outcomes correspond to the protocol (ClinicalTrials.gov Identifier: NCT00258557) |
| Other bias | Unclear | Financial assistance was provided by Janssen and Tibotec BVBA. Gilead donated Truvada (tenofovir and emtricitabine). The authors served as speakers and advisors for Bristol- Myers Squibb, Gilead, GlaxoSmithKline, Tibotec, Roche, Abbott, Boehringer Ingelheim, Vertex, Sharp & Dohme, Pfizer, Merck, Wyeth and others and have received honoraria. |

| **Risk of bias: FLAMINGO** | **Judgment** | **Rationale** |
| --- | --- | --- |
| Random sequence generation (selection bias) | Low | Randomization (1:1) was done by a central interface. Validated randomization software was used to generate the list. Randomization was stratified by HIV-1 RNA (>100000 copies per mL or ≤100 000 copies per mL) and NRTI backbone. |
| Allocation concealment (selection bias) | Low | Allocation was not concealed, but risk of bias was low as all outcomes were biomedical. |
| Blinding of participants and personnel (performance bias) | High | The trial was open-label; patients and physicians were aware of group allocations. Risk of bias was low as all outcomes were biomedical. |
| Blinding of outcome assessment (detection bias) | High | Study was open label. There is no information about the blinding of outcome assessors. |
| Incomplete outcome data (attrition bias) | Low | Loss to follow up was light (2.48%-4.1%) and investigators described it adequately. |
| Selective reporting (reporting bias) | Low | Outcomes reported conform well to trial protocol (ClinicalTrials.gov Identifier: NCT01449929) |
| Other bias | Unclear | The funders participated in study design. ViiV Healthcare participated in data collection, data analysis, data interpretation, and reviewed the report. Only 5 out of 21 authors declared not having competing interests, others were salaried employees of pharmacological companies. |

| **Risk of bias: ACTG5257** | **Judgment** | **Rationale** |
| --- | --- | --- |
| Random sequence generation (selection bias) | Low | Randomization (1:1:1) used permuted blocks stratified by HIV-1 RNA level (>100 000 vs. ≤100 000 copies/mL) with balancing by institution. |
| Allocation concealment (selection bias) | Low | Allocation was not concealed, but risk of bias was low as all outcomes were biomedical. |
| Blinding of participants and personnel (performance bias) | High | The trial was open-label; patients and physicians were aware of group allocations. Risk of bias was low as all outcomes were biomedical. |
| Blinding of outcome assessment (detection bias) | High | Study was open label. There is no information about the blinding of outcome assessors. |
| Incomplete outcome data (attrition bias) | Low | Loss to follow up was light (3.8%-5.7%) and investigators described it adequately. |
| Selective reporting (reporting bias) | Low | Outcomes reported conform well to trial protocol (ClinicalTrials.gov Identifier: NCT00811954) |
| Other bias | Unclear | Industry funding (Bristol-Myers Squibb, Gilead Sciences, Merck Sharp & Dohme Corp. Tibotec Therapeutics). Authors worked for the pharmacological companies, also received grants from them (https://www.acponline.org/authors/conflictFormServlet/M14-1084/ICMJE/M14-1084-Conflicts.pdf). |
