# Supplementary material for: Ritonavir-Boosted Darunavir Plus Two Nucleoside Reverse Transcriptase Inhibitors versus Other Regimens for Initial Antiretroviral Therapy for People with HIV Infection: A Systematic Review
Source: AIDS Res Treat. 2017 Sep 26;2017:2345617. doi: 10.1155/2017/2345617 (PMC5634582; doi:10.1155/2017/2345617)
Supplement: Supplementary file 1 — Supplement 1: PubMed search strategy, modified and adapted as needed for use in the other databases. Supplement 2: Detailed risk of bias assessment. Supplement 3: GRADE evidence profile. [file 2345617.f1.zip › mat.2345617.v2/S3_GRADE.docx]

**Supplement 3.** GRADE evidence profile

**Authors:** Balayan T, Horvath H, Rutherford GW
**Date:** 2017-01-04
**Question:** Should DRV/r (vs LPV/r) be used for initial ART in adults with HIV?
**Settings:** Argentina, Australia, Austria, Belgium, Canada, Chile, Costa Rica, Denmark, France, Germany, Greece, Guatemala, Malaysia, Mexico, Panama, Puerto Rico, Russia, Singapore, South Africa, Spain, Switzerland, Taiwan, Thailand, United Kingdom, United States of America
**Bibliography:** ARTEMIS trial

| **Quality assessment** | | | | | | | **No of patients** | | **Effect** | | **Quality** | **Importance** |
| --- | --- | --- | --- | --- | --- | --- | --- | --- | --- | --- | --- | --- |
|  |  |  |  |  |  |  |  |  |  |  |  |  |
| **No of studies** | **Design** | **Risk of bias** | **Inconsistency** | **Indirectness** | **Imprecision** | **Other considerations** | **DRV/r** | **LPV/r** | **Relative (95% CI)** | **Absolute** |  |  |
| **PVL <50 copies/mL (48 weeks)** | | | | | | | | | | | | |
| 1 | randomised trials | no serious risk of bias^1^ | no serious inconsistency | no serious indirectness | no serious imprecision | none | 254/343  (74.1%) | 226/346  (65.3%) | RR 1.13 (1.03-1.25) | 85 more per 1000 (from 20 more to 163 more) | ⊕⊕⊕⊕ HIGH | CRITICAL |
| PVL <50 copies/mL (96 weeks) | | | | | | | | | | | | |
| 1 | randomised trials | serious^2^ | no serious inconsistency | no serious indirectness | no serious imprecision | none | 271/343  (79.0%) | 246/346  (71.1%) | RR 1.11 (1.02-1.21) | 78 more per 1000 (from 14 more to 149 more) | ⊕⊕⊕O MODERATE | CRITICAL |
| **PVL <50 copies/mL (192 weeks)** | | | | | | | | | | | | |
| 1 | randomised trials | serious^2^ | no serious inconsistency | no serious indirectness | no serious imprecision | none | 236/343  (68.8%) | 198/346  (57.2%) | RR 1.2  (1.07-1.35) | 114 more per 1000 (from 40 more to 200 more) | ⊕⊕⊕O MODERATE | CRITICAL |
| **Mortality (48 weeks)** | | | | | | | | | | | | |
| 1 | randomised trials | no serious risk of bias^2^ | no serious inconsistency | no serious indirectness | very serious^3^ | none | 1/343  (0.29%) | 3/346  (0.87%) | RR 0.34 (0.04-3.22) | 6 fewer per 1000 (from 8 fewer to 19 more) | ⊕OOO LOW | CRITICAL |
| **Mortality (96 weeks)** | | | | | | | | | | | | |
| 1 | randomised trials | serious^2^ | no serious inconsistency | no serious indirectness | very serious^3^ | none | 1/343  (0.29%) | 3/346  (0.87%) | RR 0.34 (0.04-3.22) | 6 fewer per 1000 (from 8 fewer to 19 more) | ⊕OOO VERY LOW | CRITICAL |
| Mortality (192 weeks) | | | | | | | | | | | | |
| 1 | randomised trials | serious^2^ | no serious inconsistency | no serious indirectness | very serious^3^ | none | 4/343  (1.2%) | 7/346  (2.0%) | RR 0.58 (0.17-1.95) | 8 fewer per 1000 (from 17 fewer to 19 more) | ⊕OOO VERY LOW | CRITICAL |
| ≥1 SAE (48 weeks) | | | | | | | | | | | | |
| 1 | randomised trials | no serious risk of bias^2^ | no serious inconsistency | no serious indirectness | serious^4^ | none | 25/343  (7.3%) | 41/346  (11.8%) | RR 0.62 (0.38-0.99) | 45 fewer per 1000 (from 1 fewer to 73 fewer) | ⊕⊕⊕O MODERATE | CRITICAL |
| ≥1 SAE (96 weeks) | | | | | | | | | | | | |
| 1 | randomised trials | serious^2^ | no serious inconsistency | no serious indirectness | serious^4^ | none | 34/343  (9.9%) | 55/346  (15.9%) | RR 0.62 (0.42-0.93) | 60 fewer per 1000 (from 11 fewer to 92 fewer) | ⊕⊕OO LOW | CRITICAL |
| ≥1 SAE (192 weeks) | | | | | | | | | | | | |
| 1 | randomised trials | serious^2^ | no serious inconsistency | no serious indirectness | serious | none | 55/343  (16%) | 72/346  (20.8%) | RR 0.77 (0.56-1.06) | 48 fewer per 1000 (from 92 fewer to 12 more) | ⊕⊕OO LOW | CRITICAL |

^1^ Open label trial but outcome was biomedical; low susceptibility to bias. Not graded down.
^2^ Open label trial but outcome was biomedical; low susceptibility to bias. However, loss to follow-up was >20% at 96 weeks and 192 weeks. Graded down by 1.
^3^ Very few events. Optimal information size threshold not met. Graded down by 2.
^4^ Few events. Optimal information size threshold not met. Graded down by 1.

**Authors:** Balayan T, Horvath H, Rutherford GW
**Date:** 2017-01-04
**Question:** Should DRV/r (vs DTG) be used for initial ART in adults with HIV?
**Settings:** France, Germany, Italy, Romania, Russia, Spain, Switzerland, United States of America
**Bibliography:** FLAMINGO trial

| **Quality assessment** | | | | | | | **No of patients** | | **Effect** | | **Quality** | **Importance** |
| --- | --- | --- | --- | --- | --- | --- | --- | --- | --- | --- | --- | --- |
|  |  |  |  |  |  |  |  |  |  |  |  |  |
| **No of studies** | **Design** | **Risk of bias** | **Inconsistency** | **Indirectness** | **Imprecision** | **Other considerations** | **DRV/r** | **DTG** | **Relative (95% CI)** | **Absolute** |  |  |
| **PVL <50 copies/mL (48 weeks)** | | | | | | | | | | | | |
| 1 | randomised trials | no serious risk of bias^1^ | no serious inconsistency | no serious indirectness | no serious imprecision | none | 186/245  (75.9%) | 192/243  (79.0%) | RR 0.96 (0.87-1.06) | 32 fewer per 1000 (from 103 fewer to 47 more) | ⊕⊕⊕⊕ HIGH | CRITICAL |
| PVL <50 copies/mL (96 weeks) | | | | | | | | | | | | |
| 1 | randomised trials | no serious risk of bias^1^ | no serious inconsistency | no serious indirectness | no serious imprecision | none | 164/245  (66.9%) | 194/243  (79.8%) | RR 0.84 (0.75-0.93) | 128 fewer per 1000 (from 56 fewer to 200 fewer) | ⊕⊕⊕⊕ HIGH | CRITICAL |
| ≥1 SAE (48 weeks) | | | | | | | | | | | | |
| 1 | randomised trials | no serious risk of bias^1^ | no serious inconsistency | no serious indirectness | very serious^2^ | none | 13/245  (5.3%) | 26/243  (10.7%) | RR 0.5  (0.26-0.94) | 53 fewer per 1000 (from 6 fewer to 79 fewer) | ⊕⊕OO LOW | CRITICAL |
| ≥1 SAE (96 weeks) | | | | | | | | | | | | |
| 1 | randomised trials | no serious risk of bias^1^ | no serious inconsistency | no serious indirectness | serious^3^ | none | 36/245  (14.7%) | 21/243  (8.6%) | RR 1.7  (1.02-2.83) | 60 more per 1000 (from 2 more to 158 more) | ⊕⊕⊕O MODERATE | CRITICAL |

^1^ Open label trial but outcome was biomedical; low susceptibility to bias. Not graded down.
^2^ Very few events. Optimal information size threshold not met. Graded down by 2.
^3^ Few events. Optimal information size threshold not met. Graded down by 1.

**Authors:** Balayan T, Horvath H, Rutherford GW
**Date:** 2017-01-04
**Question:** Should DRV/r (vs RAL) be used for initial ART in adults with HIV?
**Settings:** United States of America (including Puerto Rico)
**Bibliography:** ACTG 5272 trial

| **Quality assessment** | | | | | | | **No of patients** | | **Effect** | | **Quality** | **Importance** |
| --- | --- | --- | --- | --- | --- | --- | --- | --- | --- | --- | --- | --- |
|  |  |  |  |  |  |  |  |  |  |  |  |  |
| **No of studies** | **Design** | **Risk of bias** | **Inconsistency** | **Indirectness** | **Imprecision** | **Other considerations** | **DRV/r** | **RAL** | **Relative (95% CI)** | **Absolute** |  |  |
| **PVL <50 copies/mL (96 weeks)** | | | | | | | | | | | | |
| 1 | randomised trials | no serious risk of bias^1^ | no serious inconsistency | no serious indirectness | no serious imprecision | none | 461/601  (76.7%) | 494/603  (81.9%) | RR 0.94 (0.88-0.99) | 49 fewer per 1000 (from 8 fewer to 98 fewer) | ⊕⊕⊕⊕ HIGH | CRITICAL |
| Mortality (96 weeks) | | | | | | | | | | | | |
| 1 | randomised trials | no serious risk of bias^1^ | no serious inconsistency | no serious indirectness | very serious^2^ | none | 13/601  (2.2%) | 6/603  (1.0%) | RR 2.17 (0.83-5.68) | 12 more per 1000 (from 2 fewer to 47 more) | ⊕⊕OO LOW | CRITICAL |
| Elevated blood bilirubin (96 weeks) | | | | | | | | | | | | |
| 1 | randomised trials | no serious risk of bias^1^ | no serious inconsistency | serious^3^ | no serious imprecision | none | 466/601  (77.5%) | 444/603  (73.6%) | RR 1.05 (0.99-1.12) | 37 more per 1000 (from 7 fewer to 88 more) | ⊕⊕⊕O MODERATE | IMPORTANT |

^1^ Open label trial but outcome was biomedical; low susceptibility to bias. Not graded down.
^2^ Very few events. Optimal information size threshold not met. Graded down by 2.
^3^ In contrast to measurement of PVL, elevated blood bilirubin is a laboratory result that does not directly inform analysis of ART efficacy. Graded down by 1.

**Authors:** Balayan T, Horvath H, Rutherford GW
**Date:** 2017-01-04
**Question:** Should DRV/r (vs ATV/r) be used for initial ART in adults with HIV?
**Settings:** United States of America (including Puerto Rico)
**Bibliography:** ACTG 5272 trial

| **Quality assessment** | | | | | | | **No of patients** | | **Effect** | | **Quality** | **Importance** |
| --- | --- | --- | --- | --- | --- | --- | --- | --- | --- | --- | --- | --- |
|  |  |  |  |  |  |  |  |  |  |  |  |  |
| **No of studies** | **Design** | **Risk of bias** | **Inconsistency** | **Indirectness** | **Imprecision** | **Other considerations** | **DRV/r** | **ATV/r** | **Relative (95% CI)** | **Absolute** |  |  |
| **PVL <50 copies/mL (96 weeks)** | | | | | | | | | | | | |
| 1 | randomised trials | no serious risk of bias^1^ | no serious inconsistency | no serious indirectness | no serious imprecision | none | 461/601  (76.7%) | 455/605  (75.2%) | RR 1.02 (0.96-1.09) | 15 more per 1000 (from 30 fewer to 68 more) | ⊕⊕⊕⊕ HIGH | CRITICAL |
| Mortality (96 weeks) | | | | | | | | | | | | |
| 1 | randomised trials | no serious risk of bias^1^ | no serious inconsistency | no serious indirectness | very serious^2^ | none | 13/601  (2.2%) | 10/605  (1.7%) | RR 1.31 (0.58-2.96) | 5 more per 1000 (from 7 fewer to 32 more) | ⊕⊕OO LOW | CRITICAL |
| Elevated blood bilirubin (96 weeks) | | | | | | | | | | | | |
| 1 | randomised trials | no serious risk of bias^1^ | no serious inconsistency | serious^3^ | no serious imprecision | none | 466/601  (77.5%) | 286/605  (47.3%) | RR 1.64 (1.49-1.8) | 303 more per 1000 (from 232 more to 378 more) | ⊕⊕⊕O MODERATE | IMPORTANT |

^1^ Open label trial but outcome was biomedical; low susceptibility to bias. Not graded down.
^2^ Very few events. Optimal information size threshold not met. Graded down by 2.
^3^ In contrast to measurement of PVL, elevated blood bilirubin is a laboratory result that does not directly inform analysis of intervention efficacy. Graded down by 1.
